# Severe Hypothyroidism and Large Goiter due to Iodine Deficiency in an Adolescent Male in the United States: A Case Report and Review of the Literature

**DOI:** 10.1155/2022/7235102

**Published:** 2022-11-02

**Authors:** Claire E. Moore, Sabitha Sasidharan Pillai, Juliana Austin, Meghan E. Fredette, Monica Serrano-Gonzalez

**Affiliations:** ^1^Department of Pediatrics, The Warren Alpert Medical School of Brown, Providence, RI, USA; ^2^Division of Pediatric Endocrinology, Hasbro Children's Hospital, Providence, RI, USA; ^3^Center for Endocrinology, Diabetes and Metabolism, Children's Hospital Los Angeles, Los Angeles, CA, USA; ^4^Department of Pediatrics, Keck School of Medicine, University of Southern California, Los Angeles, CA, USA

## Abstract

Acquired hypothyroidism due to iodine deficiency is extremely rare in the United States due to the introduction of table salt iodization in the 1920s (Leung et al., 2012). We present the case of an adolescent male with a history of mild autism spectrum disorder and an extremely restrictive diet who was found to have iodine deficiency as the etiology for his rapidly enlarging goiter and antibody-negative hypothyroidism. Thyroid-stimulating hormone (TSH) was 416 *μ*IU/mL (0.350–5.500 *μ*IU/mL), free thyroxine (T4) was <0.1 ng/dL (0.80–1.80 ng/dL), and triiodothyronine (T3) was 41 ng/dL (82–213 mg/dL) at diagnosis. The patient's 24-hour urinary iodine was undetectable. He was started on iodine supplementation with rapid visible improvement of goiter within two weeks and normalization of thyroid function tests within four weeks. Thorough dietary history and nutritional screening are important in cases of acquired hypothyroidism and/or goiter. Alternatively, diets that are low in iodized salt, dairy, bread, and seafood should raise concern for iodine deficiency, and patients with suspected or proven iodine deficiency should be screened for hypothyroidism.

## 1. Introduction

Iodine deficiency is uncommon in the United States and hence an uncommon cause of hypothyroidism. Trends in median urinary excretion of iodine as tracked by the National Health and Nutrition Examination Survey (NHANES) demonstrated that while the average individual in the United States is iodine sufficient, the median urinary iodine content has been decreasing over time. Fortunately, children's median urinary iodine excretion has been relatively stable (mean value of 190 *µ*g/L from 2011-2014 data) [2].

Because there is no public-health mandate for salt iodization in the United States, personal dietary choices determine iodine sufficiency. A transition to less iodized forms of salt and health trends encouraging decreased salt intake may be two factors contributing to a reduction in iodide intake [3]. While primary sources of dietary iodine include fish, seafood, grains, and iodized salt, children and adolescents receive 53–70% of their daily iodide intake from dairy [4]. Iodine deficiency is diagnosed by urine iodine concentrations less than 100 mcg/L [5]. Iodine is necessary for the production of thyroid hormones (T3 and T4) and its deficiency leads to hypothyroidism [6].

We report the case of a 13-year-old male presenting with a rapidly enlarging goiter who was found to have markedly elevated TSH, undetectable free T4 and low total T3 levels, and negative autoimmune thyroiditis antibodies. Iodine deficiency was identified as the ultimate etiology of his hypothyroidism. We performed a literature review and identified 6 case reports from developed nations, describing 7 children aged 7.5 months to 12 years with hypothyroidism due to iodine deficiency (Table 1) [7–12].

## 2. Case Presentation

A 13-year-old male with a history of mild autism spectrum disorder, anxiety, and attention-deficit hyperactivity disorder presented to his pediatrician with neck swelling in the setting of two months of fatigue. He had been sleeping over twelve hours per day and did not have sufficient energy to complete his schoolwork. Based on the review of family photographs, thyroid enlargement may have started about two months prior to the initial clinic visit. The pediatrician noted significant goiter. Laboratory workup revealed elevated TSH of 416 *μ*IU/mL (0.350–5.500 *μ*IU/mL), low free T4 < 0.1 ng/dL (0.80–1.80 ng/dL), and low T3 41 ng/d (82–213 ng/dL). The complete blood count was normal. Ultrasound of the thyroid was interpreted by radiology as consistent with autoimmune thyroiditis (see Table 1 for detailed description and thyroid gland dimensions); however, thyroid peroxidase (TPO) and thyroglobulin (Tg) antibodies were negative. He was referred to pediatric endocrinology for further evaluation and management.

A review of the growth charts revealed growth deceleration starting at 10 years old, with height percentiles decreasing from the 60^th^ to the 35^th^ percentile and weight percentiles declining from the 75^th^ to the 25^th^ percentile. The mother reported a very restrictive diet. He ate specific brands of bread and peanut butter, neither of which contained iodine. His diet included some chicken but no seafood, fish, dairy, or processed or canned foods. He did not consume iodized table salt. Restriction of diet was largely related to the patient's preference for certain foods in the context of his autism spectrum disorder. In addition, a year prior to the presentation, the family had eliminated dairy from the patient's diet due to presumed lactose intolerance.

**Figure 1:**
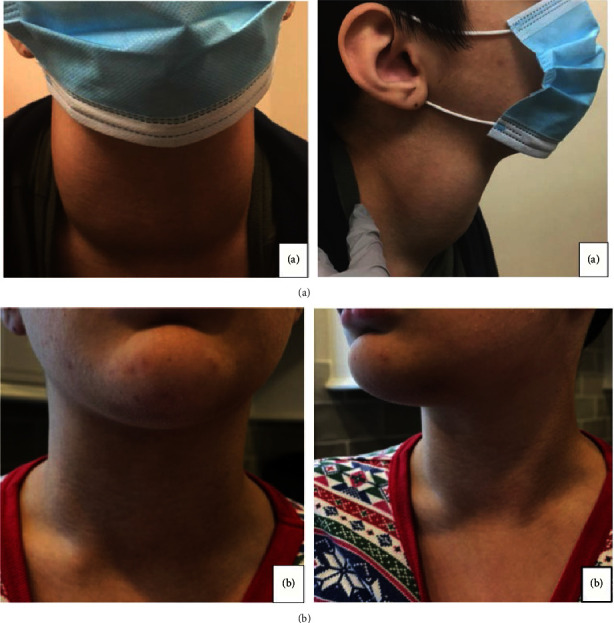
(a) Visible goiter at the initial endocrinology visit and (b) virtual follow-up two weeks after treatment initiation.

On examination, vital signs were within normal limits. Height was at the 36^th^ percentile and weight was at the 54^th^ percentile. Physical examination revealed symmetric, firm thyromegaly without distinct nodules. The right lobe measured 8 cm, and the left lobe measured 8.5 cm (Figure 1(a)).

Given his severe dietary restrictions, the possibility of iodine deficiency was considered. The patient was initially started on a low dose of levothyroxine (25 mcg daily [0.5 mcg/kg/day]) with a plan for slow, stepwise increases while his 24-hour urinary iodine was pending. Urinary iodine concentration from 24-hour collection resulted as <5.0 *µ*g/L (26–705.0 *µ*g/L), confirming severe iodine deficiency. He has been prescribed iodine supplementation of 150 mcg daily. Given his very restrictive diet, iron studies and vitamin levels were assessed. He was found to have mild iron deficiency (elevated total-iron binding capacity −505 *µ*g/dL-with normal iron, ferritin, and hemoglobin) and vitamin C deficiency (serum vitamin C undetectable at <5 *µ*mol/liter) and was started on a multivitamin with iron. Vitamins D and B12 were not deficient because he had already been receiving supplementation prior to the presentation.

Two weeks after iodine replacement, the family reported significant improvement in the size of his goiter, as well as his energy level (Figure 1(b)). Eggs were reintroduced successfully into his diet. Repeated laboratory evaluation one month after iodine initiation showed normalization of values with TSH of 0.707 *μ*IU/mL (0.350–5.500 uIU/mL) and free T4 of 1.45 ng/dL (0.80–1.80 ng/dL). The small dose of levothyroxine was discontinued following these test results, and thyroid function remained normal on subsequent follow-ups after 2 months. On follow-up examination 5 months after iodine supplementation was started, the patient had a much decreased albeit persistent goiter, with the left lobe measuring 6.5 centimeters and the right lobe measuring 6.0 centimeters.

## 3. Discussion

Iodine is a necessary component for the synthesis of T3 and T4. Initially, with mild iodine deficiency, there is upregulation of sodium-iodide symporters to increase iodine uptake into the thyroid gland, and renal iodine excretion is reduced. As the severity of deficiency worsens, there is a preferential synthesis of T3 over T4. Severe and prolonged deficiency of dietary iodine leads to a decreased production of both T3 and T4 with a subsequent appropriate elevation of TSH [5]. TSH has the secondary effect of inducing hypertrophy and hyperplasia of the thyroid follicular cells that leads to goiter [15]. Initially, goiters are smooth; however, with long-standing deficiency, patients can develop thyroid nodules which are associated with an increased risk of thyroid cancer [16].

The necessary iodine intake to maintain sufficiency varies by age. The World Health Organization recommends 90 *µ*g per day for children under 5 years, 120 *µ*g per day for children aged 6–12,150 *µ*g per day for adolescents over the age of 12 and adults, and 250 *µ*g per day for pregnant and lactating women. For our adolescent patient, a target supplementation of 100–299 *µ*g would avoid both insufficiency and excess [5].

When unacknowledged, iodine deficiency not only leads to hypothyroidism but can impair growth and cognitive development [17]. There are few modern case reports of iodine deficiency in developed nations secondary to diets restricted for autism spectrum disorder, food aversion, and food allergies (Table 1) [7–12]. All except two patients were treated with iodine supplementation. One patient was treated with kelp capsules containing 400 ug of iodine/capsule per parental request [9] and another with oral aversion was given nasogastric feeds rich in iodine content resulting in the normalization of thyroid function tests [11]. Ikomi et al. reported iodine deficiency in 85% (17/20) and acquired hypothyroidism in 33% (8/27) of patients younger than 17 years on parenteral nutrition for more than 6 months. No significant association was observed between the duration of parenteral nutrition and iodine deficiency or hypothyroidism [18].

Because the body relies exclusively on iodide ingestion through diet or supplementation to achieve iodine sufficiency, changes in personal or family dietary patterns would explain why some children are iodine deficient. In addition to health concerns about excessive salt consumption, there have been trends toward more specialized diets recently. Patients with vegan diets rely primarily on iodized table salt or foods such as seaweed for their iodine content [19] and are thus at high risk for iodine deficiency. A study on women in Boston found that a majority of vegetarians and vegans studied had not consumed iodized table salt in the last 24 hours and were not on an iodine-containing multivitamin [20].

Management of iodine deficiency involves reintroducing iodine into the diet. Thyroid function tests rapidly normalize after repletion, and levothyroxine, if started, can be discontinued. The patients' large goiters also improve quickly with near resolution noted at three-monthfollow-up visits [8, 21]. Ongoing monitoring of thyroid function tests should be performed as thyrotoxicosis can develop secondary to iodine supplementation [21].

The present case highlights the importance of thorough dietary history and nutritional screenings in cases of hypothyroidism and/or goiter. Diets that are low in iodized salt, dairy, bread, and seafood should raise concern for iodine deficiency and prompt urinary testing by 24-hour urine collection, which produces more reliable values than testing from spot urine collections [22]. Patients with suspected or proven iodine deficiency due to restrictive diets or parenteral nutrition should be screened for hypothyroidism. Repletion and maintenance of dietary iodine are expected to lead to prompt resolution of symptoms of hypothyroidism.

## Figures and Tables

**Table 1:** Reported cases of dietary restriction causing iodine deficiency hypothyroidism.

	Kanaka et al. 1992 [7]	Leniszewski et al. 2009 [8]	BrooksMJ et al. 2014 [9]	Cheetham et al. 2015 [10]	Booms et al. 2016 [11]		Yeliosof et al. 2018 [12]	Our patient
					Case 1	Case 2		
Age at diagnosis	7.5 months	12 years	9 years	4 years	5 years	2 years	23 months	13 y
Cause of dietary restriction	Developmental delay, hypotonia, and vegan diet	Autism, multiple food allergies	Eosinophilic esophagitis and food allergies	Multiple food allergies	Autism, gluten, and casein free diet	Picky eater and dairy-free diet due to eczema	Strict vegan diet	Autism
Physical examination	Goiter	Goiter	Goiter	Goiter	Goiter	Normal thyroid gland	Goiter	Goiter
TSH (*µ*IU/mL)	378 (0.3–4)	16.5 (0.5–4.5)	150.4 (0.6–4.84)	9.1 (0.3–4.7)	355 (0.47–4.53)	222.43 (0.30–5.50)	382 (0.7–5.9)	416 (0.35–5.50)
Free T4 (ng/dl)	0.2253 (0.77–2)	0.2 (0.8–2)	0.18 (0.9–1.68)	0.4894 (0.74–1.63)	0.18 (0.84–2.26)	0.24 (0.76–1.70)	<0.2 (0.85–1.75)	<0.1 (0.8–1.8)
Total T4 (ug/dl)		1.6 (4.5–10)					<0.5 (4.5–12)	
Free T3 (ng/dl)	0.4883 (0.20–0.55)	0.48 (0.34–0.48)			0.253 (0.2–0.5)			
Total T3 (ng/dl)							59(83–230)	41 (81–213)
TPO antibodies	Negative	Negative	Negative		Negative	Negative	Negative	Negative
Tg antibodies	Negative	Negative	Negative		Negative	Negative	Negative	Negative
Urine iodine (*µ*g/L)	3.03 (6.58–42.66)	<10 (100–460)	—	10.1 (50–240)	11 (100–199)	20 (100–199)	—	<5 (26–705)
Thyroid ultrasound	—	Right lobe 7.4 × 4.0 × 2.6 cm, total volume 41 cc. Left lobe 7.8 × 3.2 × 2.9 cm, total volume 38 cc	Simple goiter	Thyroid gland enlargement with hypervascularity on doppler	Enlarged hyperemic gland, suggesting Hashimoto's thyroiditis	—	Right lobe −1.5 × 1.7 × 3.3 cm, left lobe 1.2 × 1.1 × 2.4 cm	Right lobe 8.3 × 3.7 × 4.6 cm. Left lobe 8.6 × 3.2 × 4.5 cm. Heterogeneous in echotexture with a lobular contour and diffuse hyperemia

## Data Availability

The [clinical and laboratory parameters] data used to support the findings of this study are included within the article.

## Abbreviations

TSH: Thyroid-stimulating hormone
T4: Thyroxine
T3: Triiodothyronine
NHANES: National health and nutrition examination survey
TPO: Thyroid peroxidase
Tg: Thyroglobulin.
